# Some Clinical Notes on Aqua Senicula

**Published:** 1888-08

**Authors:** G. W. Sherbino

**Affiliations:** Abilene, Texas


### SOME CLINICAL NOTES ON AQUA SANICULA.

(I. H. A., June, 1888.)

G. W. Sherbino, M. D., Abilene, Texas.

After a year's experience, the following clinical symptoms have been collected; the potencies used were 10M, 50M [Skinner] and MM, CMM, DMM [Swan].

Mind.—Irritability. Weakness of memory; inability to think; cannot find the word to express an idea. Low-spirited, despondent; aversion to company, desires to be alone. Disinclination to talk. Child throws head backward, when being nursed, especially if displeased. The irritability is a very prominent symptom.

Inner Head.—Pain commences in forepart of left side of head passing back to the left mastoid process; it is aggravated by motion, in warm room, from a misstep or jar, by noise; is relieved at once by putting the hands in cold water. A peculiarity of the head symptoms is that the pain was felt mostly on the right side of the head; if he put his fingers on the left side of the head the pain would dart to that part. Thus he could draw the pain to any part of the head he placed his fingers upon; he could draw it to the lower jaw by so touching it. Head relieved in a cool room, in cool air; the wind makes her chilly but it relieves the head. [50M, Skinner.]

Occipital headache, pains all night; spreads from the occiput like a flash, extending to the front, face, and head; had to hold the occiput in her hands, sitting on a low bed, worse when lying. Pain extending from her head to the stomach. [From stomach to head, Lith-carb.] When the pain went to the stomach she became deathly sick, retching or vomiting. Headache so severe she nearly lost her reason, groaning with the pain aggravated by efforts at vomiting. Pain over orbital region and the eyes.

OUTER HEAD.—Sweats on the back of the head when sleeping; child sweats on back of neck as soon as it begins to nurse, cold and clammy.

EYES.—Blepharitis, worse from taking cold or from studying too much. Itching of the eyelids, with scurf forming, white and scaly, at the roots of the hairs. Ameliorated by cold water or cool fresh air. There was redness of the ocular and palpebral conjunctiva when the exacerbations came on [chronic cases]. Ulceration of the cornea with ophthalmia; swelling of the lids, better from cold; aggravation from sunlight or lamp-light and better from shading the eyes. Pimples and pustules around the eyes, in ophthalmia.

EARS.—Sores behind the ears, with a clear sticky discharge; sticks the ears to the side of the head. Redness in front of and around the ear; scabs with pus under them; swelling of the parts, aggravated by any covering, which caused increased heat; child scratches when it gets warm in bed; ameliorated by uncovering or by exposure to cool air. Sensation as of cold air blowing on the ears. Had to keep cotton in the ears to keep the air out; has to keep the head wrapped up. Pain shoots up from the neck into the left ear, making her jump and cry out.

FACE.—Neuralgia of the face, anæmic, pale; aching in face extending to the tip of the nose. Large acne spots all over the face and forehead, filled with matter; disfiguring the boy's face; one crop succeeding the other.

TEETH.—Pain in the right upper incisor, which had been plugged with gold one year ago. Tooth feels extremely sore and as if too long; dull aching and at times pulsating; feels congested and as if pricking the gums would relieve. Boring pain extending to the bones of the nose around the right eye, into the temple and forehead. Gumboil on the alveolar process. Aggravated by contact, from touching by the tongue, from compressing the upper lip, from chewing, from motion. Feels as if ulcerated at the root; ameliorated from lying down, from sleeping. The pain commences again in the morning as soon as he awakes and opens the eyes, on motion. Pain starts in the tooth in front of the wisdom tooth, extending up the side of the face to the malar bone into the temple, there it throbbed and beat severely; aggravated at night from lying down; pain comes in paroxysms, coming and going suddenly. Shooting pain along the jaw on the left side, even in the jaw that had false teeth.

TONGUE.—Sudden filling of the sub-lingual glands until about a half teaspoonful of salty saliva would be discharged; so salty could not swallow it.

THROAT.—Constant desire to clear the throat; constant hawking, worse in the morning after rising, better in the fresh air. Dropping of mucus into the throat [in naso-pharyngeal catarrh].

STOMACH.—Increased appetite, gets very hungry before meals, relieved by eating; bloating of the stomach after eating. Nausea, retching, and vomiting of glairy mucus [with headache].

ABDOMEN.—Lumbo-abdominal neuralgia; has been suffering for a week with pain in the right side of the lumbar region going around over the right side of the abdomen, so severe that he could not rest day nor night; worse from motion; pain in paroxysms. He extended all his fingers and then suddenly grasped with them, saying that is the way the pain takes me. Laid on his back, left foot and leg elevated, the leg flexed; the right leg resting on the left. Touching the abdomen brought on the pain.

STOOLS.—Diarrhœa night and day; thin, watery, and offensive. Before stool, pain in the hypogastric region with urging; after stool pain in hypogastric is relieved. Stool resembling scrambled eggs; offensive-smelling like rotten cheese [also Bry., Hepar]; the odor of it is very penetrating; it goes from room to room; though the child is kept clean the odor of its stools clings to it. Child constipated from birth; stool almond shaped; bowels moved every second or third day; terrible straining, so severe that tears came out of the child's eyes, and face so red it looked as if it would burst.

Female Sexual Organs.—Sweat about a week before the menses come on, lasts during the menses. Leucorrhœa before and during the menses; leucorrhœa is milky, is worse from a jar or stepping heavily on the floor in the morning after getting out of bed; is worse on commencing to move after having been quiet. Menses a week too soon or a week too late. Before menses is despondent and low-spirited, relieved after the flow begins. Burning in the vagina and uterus ameliorated by cold water injections. Leucorrhœa yellow, aggravated walking or standing, during stool. Prolapsus uteri worse when standing with leucorrhœa; burning in the vagina, relieved by cold water injections. Menses pale, watery; sweats so profusely during the menses the bed feels wet; sweats on the upper parts.

Larynx.—Sensation as if a cold, wet string were tied around the front of the larynx, causing a choking sensation [with headache].

Chest.—Sensation of a weight on the chest, causing difficulty of breathing [menstruation].

Neck.—Sensation as of a cold, wet string tied around the front of the neck, causing a choking feeling; cold, clammy feeling on the back of the neck; pillow wet at night [from sweat?]; stitching pain on the right side of the neck, worse when turning the head; sensation of enlargement of the back of the neck [also Rhus-t. and Sepia].

Upper Limbs.—Pain from the right shoulder to the back of the head; pain from the point of the left shoulder along the clavicle to the sternum; aching in different parts of the body, now in the shoulder, then changing to some other part; pain worse in the joints. A peculiarity of these pains is that wherever they may be the part is covered by a cold sweat. Pain commences between the shoulders and passes up the back of neck into the head; pain commencing in each hand and fingers, passing up both arms to meet at centre of chest or sternum; sometimes the pain begins in the shoulders and goes downward to the hands and fingers; it is a dull aching with a cold sensation, as is experienced before freezing or a sensation as felt from holding a piece of ice on a part; is aggravated in the morning from motion; is ameliorated by lying down about fifteen minutes. Tingling in the hands and fingers, aggravated on putting the hands in warm water; when washing the hands in warm water she has to take them out and let them cool, they tingle so. The feet have this same tingling. Any excitement brings on this tingling; it is aggravated when the cold sensation comes on; the hand pains as if she had been holding ice in it. When the spells come on she gets stupid and sleepy; after the pains gets very hungry; at the height of the pain she gets relief from eating; the pain is worse when the stomach is empty. After the pain subsides there is sweat on the face, forehead, and back of the neck, cold and clammy as if she had had a chill; aching at the middle of right arm between the shoulder and elbow, as if in the bone; relieved by compressing the arm with the left hand.

LOWER LIMBS.—Coldness of the feet and legs, which felt like ice [in parturition]; so cold I could not bear to have my uncovered arm come in contact with her cold limbs; cold, clammy feet, the soles feel sticky, as though she had stepped into molasses; the stockings stick to the soles of the feet.

NERVES.—Restlessness and nervousness; child cannot be still a moment, has to be in constant motion even during sleep; weak and weary, good for nothing, wants to rest all the time.

SLEEP.—Child restless, tosses about all the night; drinks every few minutes, a little at a time.

FEVER, SWEAT, ETC.—Skin moist; sweat about the head; cold and clammy on back of the neck; cold, clammy hands and feet, does not want to be covered. Chill followed by heat and sweat on part laid on, or where the limbs cross sweat between them; sweat on part not laid on; sweat on falling asleep; child sweats on beginning to nurse on the forehead, on the face, and on the back of the neck, wetting the mother's arm.

TEMPERATURE AND WEATHER.—Patient is worse in damp weather; cool, fresh air relieves the headache either when riding or walking; worse in summer when the days begin to get very hot.

SENSATIONS.—As of cool air blowing into the ears; as if the teeth were too long and extremely sore at their roots; as if pricking the congested gums would relieve the toothache; as if clutched by the fingers, in the right side of the abdomen; as if a cold, wet string were tied around the neck, causing a choking sensation [with headache]; as if the teeth had been pulled and then left in their sockets; as of a weight on the chest, causing difficult breathing [menstruation]; cold sensation on the back of the neck [Silicea]; as if the neck were enlarged; as if the hands and arms had been exposed to a temperature of thirty degrees below zero; of tingling of fingers and hands, also of feet, as if she had been handling ice; as if the feet had been in molasses.

TISSUES.—Skin and muscles lax; paleness of skin of body; veins appear blue through the transparent skin; red blood corpuscles seem to be deficient; wasting of body, most marked in neck and thighs [marasmus], following diarrhœa; hives all over the body; intolerable itching at night; child suffering with prickly heat; fine red rash all over the body, itching terribly; sweats at night on the back of the neck, cold and clammy.

RELATIONSHIP, *Antidotes.*—Calcarea, Lycopodium, Podophyllum, and Phosphoric acid.

*Compare with:* Acon., Ars., Alum., Bell., Borax, Calc., Camph., Graph., Lyc., Natr-m., Podoph., Phos-ac., Psor., Puls., Secale, Sil., Sulph. With Ars. and Cina [thirst and restlessness]; Aloe, Lyc., Phos-ac. [in diarrhœa]; Acon. [tingling of hands and fingers]; Alum. [prolapsus and leucorrhœa]; Bell., Calc., Sil. [sweat about the head in fever]; Bry. [motion]; Actea, Puls. [headache better in the cool air]; Graph., Mezer., Sulph. [skin, sore behind the ears]; Lyc. [bloating after meals]; Natr-m., [chill, fever, and sweat]; Aur., Caust., Iod., Graph., Kali, Oleand., Rhus [aggravation from hunger].

